# Heterogeneous and efficient transesterification of *Jatropha curcas* L. seed oil to produce biodiesel catalysed by nano-sized SO_4_^2−^/TiO_2_

**DOI:** 10.1098/rsos.181331

**Published:** 2018-11-07

**Authors:** Chao Chen, Lei Cai, Xinchen Shangguan, Liang Li, Yanping Hong, Guoqiang Wu

**Affiliations:** 1 School of Food Science and Engineering, Jiangxi Agricultural University, Nanchang, Jiangxi 330045, People's Republic of China; 2 Jiangxi Provincial Food and Drug Administration, Nanchang, Jiangxi 330029, People's Republic of China

**Keywords:** biodiesel, *Jatropha curcas* L. seed oil, transesterification, SO_4_^2−^/TiO_2_, heterogeneous

## Abstract

Developing high-efficiency hetero-catalysts for transesterification reaction is of great importance in the production of biodiesel from *Jatropha curcas* L. seed oil (JO). Here, we synthesized a series of sulfated TiO_2_ by treating with varying H_2_SO_4_ concentration (SO_4_^2−^/TiO_2_) and TiO_2_ catalysts and applied to the transesterification of JO. Furthermore, these heterostructures were characterized by many characterization methods including XRD, FT-IR, N_2_-adsorption, SEM, TEM, TG, py-IR and NH_3_-TPD, and their catalytic performance was investigated under various operating conditions. The results reveal that both the Brønsted and Lewis acid sites are presented in the SO_4_^2−^/TiO_2_ catalysts, while only Lewis-type sites are observed in the TiO_2_ catalyst. And the acid intensity, surface area and mesoporous volume of catalysts are improved obviously after treating TiO_2_ with sulfuric acid. Then the SO_4_^2−^/TiO_2_ catalysts exhibit much higher catalytic activity than TiO_2_ catalyst, which is attributed to the larger surface area and mesoporous volume and stronger acidity. Furthermore, the reusability behaviour of 1.5 SO_4_^2−^/TiO_2_ catalyst in the transesterification of JO was also studied.

## 1. Introduction

**Scheme 1.**
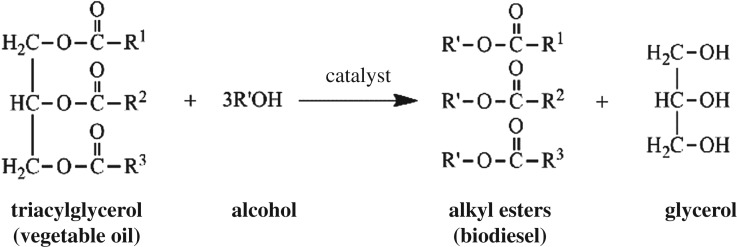
Reaction processes in the transesterification of VO [12].

The consumption of finite fossil fuels (e.g. petroleum, coal and natural gas) constantly and rapidly increases due to the course of industrialization and population growth. However, there are still many deficiencies of these non-renewable sources, such as high costs, lowly efficiency and environment unfriendly [1–6]. Therefore, the research effort focused on alternative sources of highly efficient, green and renewable energy has become very popular in recent years. Biodiesel, the monoalkyl esters of long chain fatty acids (C_12_–C_22_) derived from a renewable lipid feedstocks such as vegetable oil (VO) or animal fat, is providing a substitute or additive to diesel as a kind of alternative energy in developing as well as developed countries [7–10]. Usually, vegetable oils are preferred to be used as the feedstock due to its low cost and simple method in the production process of biodiesel [3,5,11]. As shown in Scheme 1, the biodiesel can be prepared by transesterification process combining VO with alcohol in the presence of the catalyst to form fatty acid alkyl esters (i.e. biodiesel) and glycerol [4,12].

*Jatropha curcas* L. seed oil (JO), as a typical non-edible oil, which is one of the best-suited feedstocks for biodiesel production in terms of economical, sociological and environmental implications [3,13]. Therefore, many researchers have paid great attention to the studies on the production of low-cost biodiesel from JO. Generally, catalytic transesterification of JO can be carried out over homogeneous [14–16] or heterogeneous catalysts [17–22]. Unfortunately, the homogeneous catalysts exhibit the fast reaction rate and high conversion, but the removal and reusability of catalysts are difficult and producing a large amount of hazardous wastewater. To overcome the above drawbacks, heterogeneous catalysts are the preferred catalysts which allow the catalysts to be easily separated and re-used. Furthermore, heterogeneous catalytic transesterification is divided into base catalysed [17,23,24], acid catalysed [18,25,26] and enzymatic catalysed [21,27–29]. Clearly, the solid base is not a suitable catalyst for biodiesel production because of the high free fatty acid (FFA) content of JO, which will result in soap formation [30,31]. Additionally, enzymatic transesterification has severe disadvantages of high costs, long residence times and poor stabilities, although it is more efficient, highly selective and involves less energy consumption [2,3,25,31]. However, heterogeneous acid catalysis can be a good way to produce the biodiesel using the solid acid catalysts, such as H-form zeolites [32,33], carbon-derived materials [26,34], heteropolyacids [30,35], acidic ion-exchange resins and mesostructure silica [36,37], etc.

As well known, the sulfated metal oxides (e.g. SO_4_^2−^/ZrO_2_, SO_4_^2−^/Nb_2_O_5_ and SO_4_^2−^/TiO_2_) are typical solid super acid, and they possess potential economic and green benefits for a wide variety of hydrocarbon reactions [4,13]. Hence, we think that the sulfated metal oxide catalyst can also exhibit good catalytic activity in the transesterification of JO. Actually, sulfated metal oxides have been applied in esterification and transesterification reaction and shown good catalytic performance in catalytic reaction systems [16,38,39].

The objective of this work is to develop and test sulfated metal oxide catalysts in the transesterification of JO. Firstly, a series of sulfated TiO_2_ treated with different H_2_SO_4_ (SO_4_^2−^/TiO_2_) and TiO_2_ catalysts have been prepared and characterized carefully by multiple techniques. Furthermore, particular focus has been given to the effect of H_2_SO_4_ concentration of the SO_4_^2−^/TiO_2_ catalysts on the transesterification reaction, and the operation conditions, such as reaction time, CH_3_OH : JO (molar ratio), catalyst content and reaction temperature, were also systematically investigated. Additionally, the reusability behaviour of sulfated TiO_2_ catalyst in this reaction was also studied.

## 2. Material and methods

### 2.1. Materials

*Jatropha curcas* seeds were supplied by Lv You Seed of Shuyang Co. Ltd, China. Titanium tetrachloride (TiCl_4_) and methyl heptadecanoate were purchased from Aladdin Chemistry Co. Ltd, China. Fatty acid methyl ester standards were purchased from Nu-Chek-Prep, Inc., USA. Ammonia solution (28 wt% in H_2_O) was purchased from Chinasun Specialty Products Co. Ltd, China. Sulfuric acid (H_2_SO_4_) and hexane were purchased from Tianjin Yongda Chemical Reagent Co. Ltd, China. Methanol and petroleum ether were purchased from Tianjin Damao Chemical Reagent Factory, China.

### 2.2. Synthesis of catalyst samples

The TiO_2_ was prepared according to the literature with modification and carried out in a 500 ml three-neck round-bottom flask with mechanical stirring and the following procedure [40]. An amount of TiCl_4_ (10 ml) was slowly added to deionized water (200 ml) and cooled by ice water. Ammonia solution was then added dropwise to the above mixture to a final pH around 8.0 ∼ 9.0. The precipitate was filtered, washed with deionized water until no Cl^−^ (tested by 1.0 mol l^−1^ AgNO_3_ solution) and dried at 393 K for 12 h. The obtained solids (Ti(OH)_4_) were powdered below 100 mesh, then calcined at 823 K for 3 h in air to obtain the TiO_2_ sample.

The SO_4_^2−^/TiO_2_ was prepared by the impregnation of the above Ti(OH)_4_ with 0.5 mol l^−1^ H_2_SO_4_ under stirring for 12 h. Afterwards, the mixture was filtered, washed with deionized water until no SO_4_^2−^ (tested by 1 mol l^−1^ BaCl_2_ solution), dried at 393 K for 12 h and finally calcined at 823 K for 3 h, as synthesis sample denoted as 0.5SO_4_^2−^/TiO_2_. Similarly, the other SO_4_^2−^/TiO_2_ samples are denoted as SO_4_^2−^/TiO_2_, with *x* varied at 1.0, 1.5 and 2.0, respectively.

### 2.3. Catalyst characterization

X-ray powder patterns (XRD) were obtained with a RIGAKU Ultima IV diffractometer using Cu K*α* radiation. Nitrogen physisorption (N_2_-adsorption) measurements were carried out on a Micromeritics ASAP 2020 M apparatus at the temperature of liquid nitrogen (77 K). The specific surface area and pore volume were calculated according to the Brunauer–Emmett–Teller (BET) and Barrett–Joyner–Halenda (BJH) methods, respectively. Fourier-transform infrared (FT-IR) spectra of the catalyst samples were recorded on a Thermo Nicolet iS5 FT-IR spectrometer (KBr pressed flake). The acidity of the catalysts was measured using temperature-programmed desorption of ammonia (NH_3_-TPD) on a Micromeritics ASAP 2920 instrument. The acid properties of the catalysts were also investigated by pyridine FT-IR (Py-IR), performed on a Bruker TENSOR 27 instrument equipped with an *in situ* reactor cell. The samples were pre-treated firstly, the system was then degassed and evacuated at designated temperature, and the IR spectra were recorded. Scanning electron microscopy (SEM) images were recorded on a SUPRA55 apparatus. Transmission electron microscopy (TEM) images were obtained on a JEM-2100F microscope. Thermo-gravimetric analyses (TG) of the samples were carried out on an SDT Q600 apparatus from 298 to 1073 K with a heating rate of 10 K min^−1^ in air (25 ml min^−1^).

### 2.4. Preparation of JO

*Jatropha curcas* seeds were put in a desiccation oven and dried at 393 K for 24 h. They were then chopped using a grinder to a size of 80 meshes. Extracting the *Jatropha curcas* L. seed oil (JO) with solvents was carried out in a 500 ml three-necked round-bottom flask fitted with a reflux condenser under vigorous stirring. In a case, 40 g *Jatropha curcas* seeds powder and 250 ml of hexane (solvent) were used. The extraction process was heated at 343 K for 5 h. A rotavapor was used at 348 K for 25 min in order to remove the solvent. Finally, the JO was dried at 333 K until its weight was consistent. And the acid value and FFA content of the JO are 15.30 mg_KOH_ g^−1^_oil_ and 7.65% w/w, respectively.

### 2.5. Transesterification of JO

**Figure 1.**
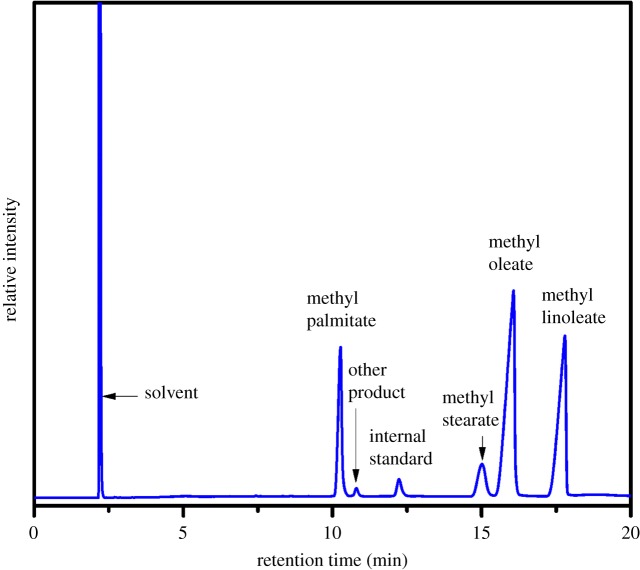
Representative gas chromatograms obtained from biodiesel samples produced from JO.

Transesterification of JO was carried out in a 25 ml PTFE-lined stainless steel autoclave under vigorous stirring, and the reaction temperature was achieved on a hot-plate stirrer (MR Hei-Tec, Heidolph, Germany) connected to an electronic temperature controller (EKT 3001, Reax 2, Heidolph, Germany) oil bath. In a typical experiment, the reaction mixture containing 3.0 g of JO, 1.0 g of methanol, 1.0 g of petroleum ether and 0.12 g of catalyst was added in the reactor. Then the reaction system was heated to 393 K and kept constant for 24 h under the stirring rate of 600 r.p.m. After the end of the reaction, the final solution was separated from the catalyst by centrifugation. The liquid phase obtained was kept in a separating funnel to separate the upper organic layer, containing the target product (biodiesel), and finally rotary-evaporated to yield the biodiesel. The catalytic performance of the catalyst material in transesterification was tested with JO. The concentrations of fatty acid methyl esters (FAMEs) were analysed on a gas-chromatographic instrument (Agilent 7890B, GC) equipped with an FID detector and FFAP capillary column (30 m × 0.32 mm × 0.50 µm) with high purity nitrogen (99.99%) as the carrier gas. Analytical conditions were as follows: injector port at 493 K, FID detector at 513 K and the oven temperature was programmed from 423 K at 15 K min^−1^ to 483 K (16 min). Methyl heptadecanoate was applied as an internal standard [41]. After transesterification of *Jatropha curcas* L. seed oil, the composition of FAMEs was determined by GC analysis, which was as follows (wt%): methyl palmitate = 14.6%, methyl stearate = 7.6%, methyl oleate = 44.5%, methyl linoleate = 32.5%, other products = 0.8%, which is very close to that result reported in the reference [42]. And the detail GC graph of biodiesel from JO is shown in figure 1.

Biodiesel (FAMEs) was the main target product. X_JO_, S_MP_, S_MS_, S_MO_, S_ML_ and S_Biodiesel_ denote the conversion of JO (JO conv.) and the selectivity of methyl palmitate, methyl stearate, methyl oleate, methyl linoleate and biodiesel (biodiesel sel.), respectively. These key parameters were calculated as follows:

2.1 $$X_{\mathrm{JO}}=\frac{n_{\mathrm{JO}}^{0}-n_{\mathrm{JO}}}{n_{\mathrm{JO}}^{0}}\times 100\text{\%},$$

2.2 $$S_{\mathrm{MP}}=\frac{n_{\mathrm{MP}}}{n_{\mathrm{MP}}+n_{\mathrm{MS}}+n_{\mathrm{MO}}+n_{\mathrm{ML}}+n_{\mathrm{Other}}}\times 100\text{\%},$$

2.3 $$S_{\mathrm{MS}}=\frac{n_{\mathrm{MS}}}{n_{\mathrm{MP}}+n_{\mathrm{MS}}+n_{\mathrm{MO}}+n_{\mathrm{ML}}+n_{\mathrm{Other}}}\times 100\text{\%},$$

2.4 $$S_{\mathrm{MO}}=\frac{n_{\mathrm{MO}}}{n_{\mathrm{MP}}+n_{\mathrm{MS}}+n_{\mathrm{MO}}+n_{\mathrm{ML}}+n_{\mathrm{Other}}}\times 100\text{\%},$$

2.5 $$S_{\mathrm{ML}}=\frac{n_{\mathrm{ML}}}{n_{\mathrm{MP}}+n_{\mathrm{MS}}+n_{\mathrm{MO}}+n_{\mathrm{ML}}+n_{\mathrm{Other}}}\times 100\text{\%}$$

2.6 $$\;\mathrm{and}\;S_{\mathrm{Biodiesel}}=S_{\mathrm{MP}}+S_{\mathrm{MS}}+S_{\mathrm{MO}}+S_{\mathrm{ML}}$$

where $n_{\mathrm{JO}}^{0}$ and *n*_JO_ denote the initial and final molar content of JO, respectively. *n*_MP_, *n*_MS_, *n*_MO_, *n*_ML_ and *n*_Other_ represent the mole content of methyl palmitate, methyl stearate, methyl oleate, methyl linoleate and other products, respectively. In all the experiments, the amounts of other products are too low to be measured, so they were not shown in tables and figures.

## 3. Results and discussion

### 3.1. Characterization results of the catalyst samples

**Figure 2.**
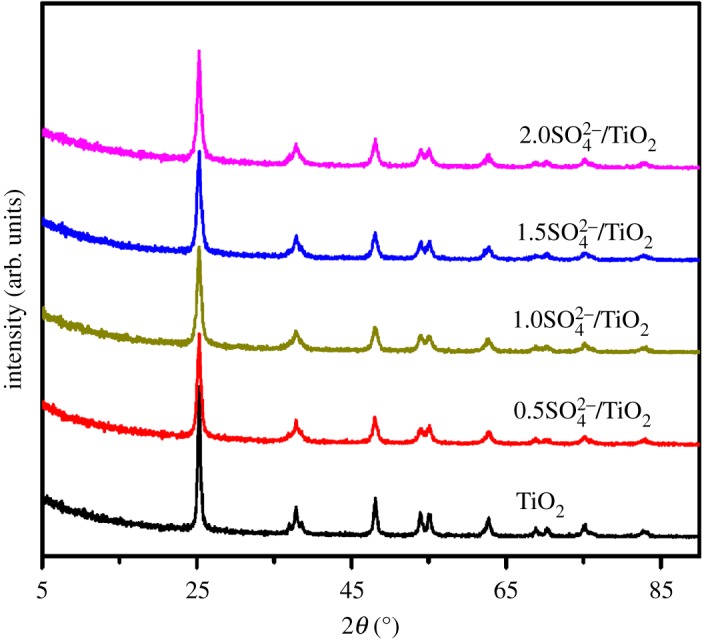
XRD diffraction patterns of the TiO_2_ and SO_4_^2−^/TiO_2_ samples.

**Figure 3.**
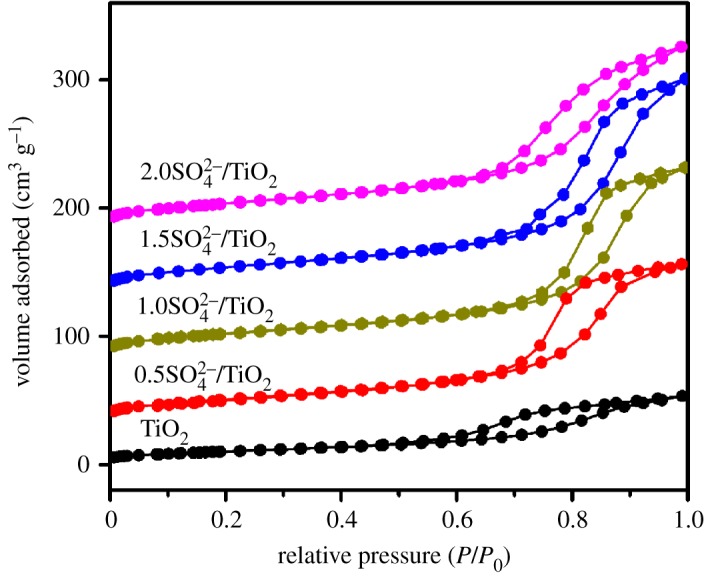
Nitrogen physisorption curves of the TiO_2_ and SO_4_^2−^/TiO_2_ samples.

Figure 2 shows the XRD patterns of TiO_2_ and SO_4_^2−^/TiO_2_ (*x* is 0.5, 1.0, 1.5 and 2.0) calcined at 823 K for 3 h. The typical diffraction pattern of TiO_2_ clearly indicates that the sample is pure anatase phase (JCPDS Card No. 21–1272) [43]. Additionally, the similar characteristic peaks at 2*θ* of 25.3°, 37.9°, 48.0°, 54.0°, 55.1° and 62.7° are observed in the SO_4_^2−^/TiO_2_ samples, indicating that the structures of these samples have no differences [40,44]. The N_2_ adsorption–desorption isotherm and NH_3_-TPD measurements of all samples are shown in figure 3 and table 1, respectively. The adsorption and desorption isotherms of TiO_2_ and SO_4_^2−^/TiO_2_ samples show type IV behaviour with the typical hysteresis loop, suggesting narrow slit-shaped pores that are generally associated with plate-like particles. And these existing pores are ascribed to the aggregation of single crystals [43]. In addition, the results of the specific surface area and pore volume calculated with the BET and BJH method, respectively, indicated that the TiO_2_ and SO_4_^2−^/TiO_2_ samples are mesoporous structures without any micropore. However, the SO_4_^2−^/TiO_2_ samples exhibit much larger surface area and mesopore volume than TiO_2_ sample, and the surface area increases with the impregnation sulfuric acid content, which is in agreement with the results obtained in the previous reference [45]. This may be because the TiO_2_ particles are partially dissolved by H_2_SO_4_, which would reduce the particle size and lead to the increase in BET area [46]. Similarly, the mesopore volume, which is the hole between the crystal particles, will increase when the catalyst particle size decreases in these SO_4_^2−^/TiO_2_ samples, in good agreement with the results obtained by nitrogen sorption (figure 3).

**Table 1.** The specific surface areas, pore volumes and NH_3_-TPD measurements of all catalysts.

sample	S_BET_ (m^2^ g^−1^)	*V*_Mesopore_ (cm^3^ g^−1^)	*V*_Micropore_ (cm^3^ g^−1^)	total NH_3_ desorbed (mmol g^−1^)
TiO_2_	33.8	0.08	0	0.16
0.5SO_4_^2−^/TiO_2_	70.4	0.19	0	0.33
1.0SO_4_^2−^/TiO_2_	74.7	0.23	0	0.38
1.5SO_4_^2−^/TiO_2_	80.9	0.26	0	0.39
2.0SO_4_^2−^/TiO_2_	81.2	0.23	0	0.37

**Figure 4.**
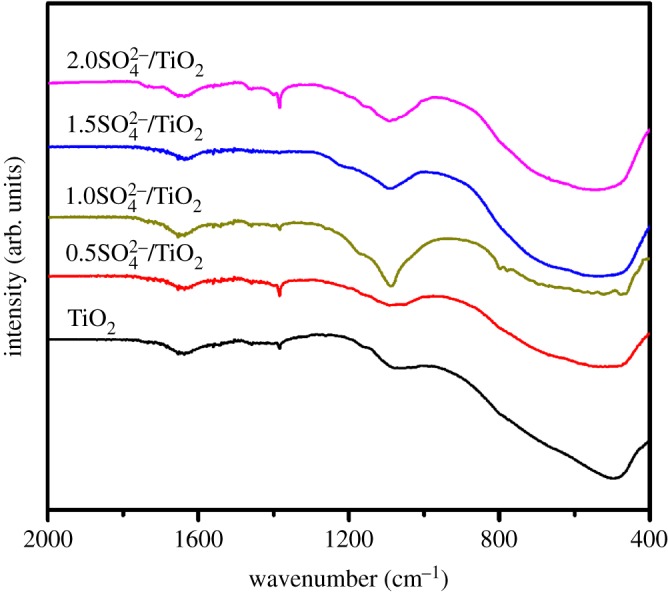
FT-IR spectra of the TiO_2_ and SO_4_^2−^/TiO_2_ samples.

**Figure 5.**
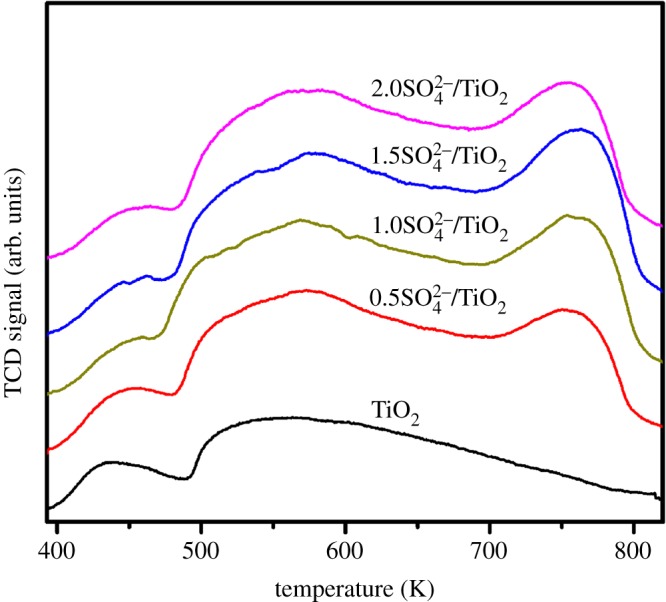
NH_3_-TPD spectra of the TiO_2_ and SO_4_^2−^/TiO_2_ samples.

**Figure 6.**
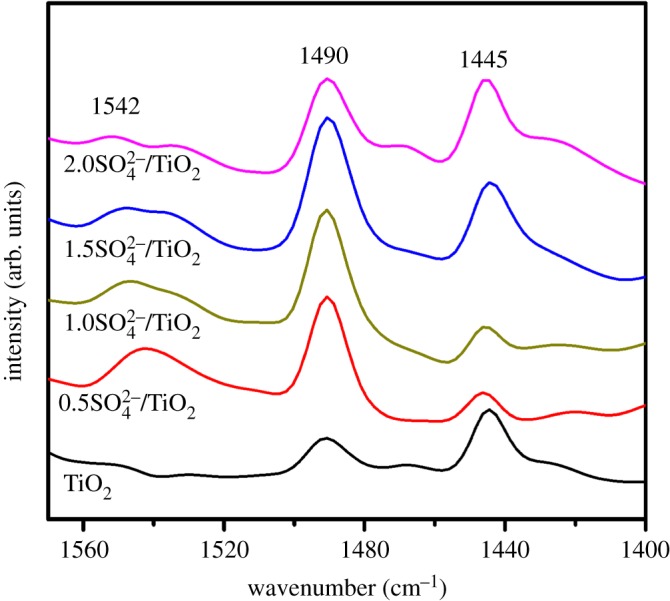
Py-IR spectra of the TiO_2_ and SO_4_^2−^/TiO_2_ samples after degassing at 473 K.

**Figure 7.**
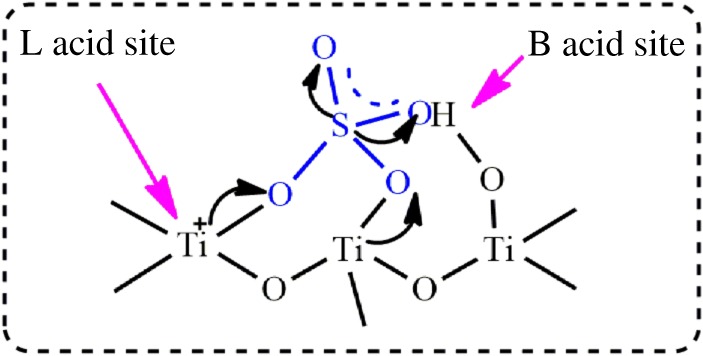
Illustration of the Brønsted and Lewis acid sites in SO_4_^2−^/TiO_2_ catalyst.

The FT-IR spectra of all samples are given in figure 4. The broad peak at about 500 cm^−1^ belongs to Ti–O stretching vibration. As for the SO_4_^2−^/TiO_2_ samples, the absorbance bands at 1177 cm^−1^ demonstrate that the SO_4_^2−^-dopant might coordinate with TiO_2_ in the network [47,48]. And these samples exhibit obvious absorption at 1640 cm^−1^, corresponding to the stretching vibration of the hydroxyl group on the surface [49]. As we know, although TiO_2_ itself possesses the acidity, the acidity of TiO_2_ can be further enhanced by modifying its surface with sulfate groups [50]. The NH_3_-TPD spectra of TiO_2_ and SO_4_^2−^/TiO_2_ with different impregnation sulfuric acid concentration samples are shown in figure 5 and table 1. The peaks shown in these profiles are assigned to the desorption of NH_3_ from the acid sites of the sample surface. Desorption temperature is related to the acid strength of SO_4_^2−^/TiO_2_, and the higher desorption temperature, the stronger acid strength [51]. There are three principal desorption peaks in the range of 390–450, 530–590 and 720–790 K. And these peaks correspond to the weak acid sites, middle acid sites and strong acid sites, respectively [52]. It can be obviously seen that TiO_2_ contains some weak acids and middle acids. The acidity of SO_4_^2−^/TiO_2_ is stronger than that of TiO_2_ after treatment of sulfuric acid. And the laws remain static that more middle acids and strong acids between SO_4_^2−^ and TiO_2_ form with the increase in sulfuric acid concentration. And these stronger acids can promote the transesterification reactions [3,4]. The acid type of the solid samples is further determined by using pyridine-IR technology (figure 6). The peaks around 1542 and 1445 cm^−1^ can be assigned to the adsorption of coordinated pyridine in Brønsted (B) acid sites and Lewis (L) acid sites, respectively, and the peak at 1490 cm^−1^ is ascribed to a combination of B and L acid sites [53]. The results show all the samples contain an amount of L acid sites, which may result from the coordination between SO_4_^2−^ and TiO_2_ in the network [48]. However, the Brønsted acidic sites are formed in the SO_4_^2−^/TiO_2_ but are not presented in TiO_2_ sample, attributing to the surface adsorption between SO_4_^2−^ species and TiO_2_. As shown in figure 7, in one SO_4_^2−^/TiO_2_ unit, two oxygen atoms from S–O bonds are bonded to Ti atoms in addition to the coordination of an S = O group with a Ti atom, and the acidic proton is derived from the surface hydroxyl group of TiO_2_ induced by the sulfate group. This acidic proton can be released easily owing to three S–O–Ti bonds linking one SO_4_^2−^ group with the TiO_2_ matrix, which results in the Brønsted acidic strength of the SO_4_^2−^/TiO_2_ [4]. That is to say, the sulfated TiO_2_ samples exhibit stronger acidity than pure TiO_2_, which is in good agreement to the experimental results of NH_3_-TPD. And the stronger acidity of the solid acid catalysts could promote the transesterification reactions [4].

**Figure 8.**
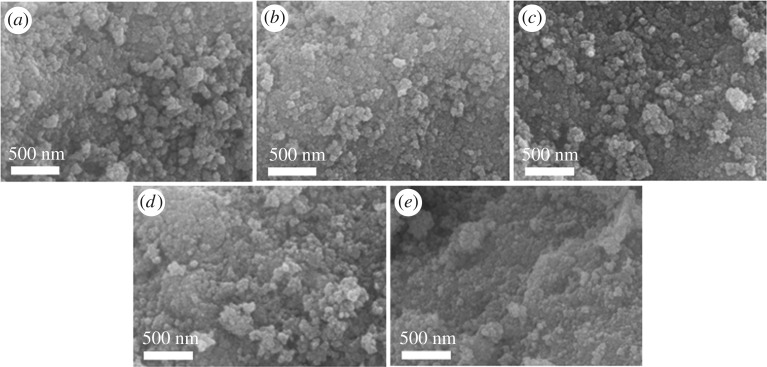
SEM images of all samples: (*a*) TiO_2_, (*b*) 0.5SO_4_^2−^/TiO_2_, (*c*) 1.0SO_4_^2−^/TiO_2_, (*d*) 1.5SO_4_^2−^/TiO_2_, (*e*) 2.0SO_4_^2−^/TiO_2_.

**Figure 9.**
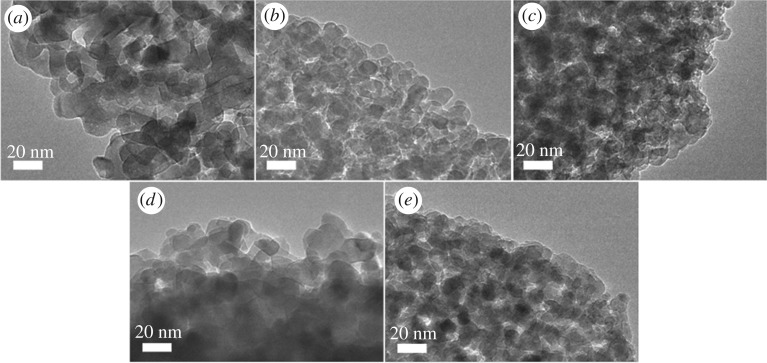
TEM images of all samples: (*a*) TiO_2_, (*b*) 0.5SO_4_^2−^/TiO_2_, (*c*) 1.0SO_4_^2−^/TiO_2_, (*d*) 1.5SO_4_^2−^/TiO_2_, (*e*) 2.0SO_4_^2−^/TiO_2._

The texture and morphology of catalysts are very important parameters and may influence the catalytic activity. The SEM images of pure TiO_2_ and SO_4_^2−^/TiO_2_ are shown in figure 8. As shown in figure 8*a*, the SEM image of the TiO_2_ depicts that the particles are in the form of aggregates and the surface is irregular. The SO_4_^2−^/TiO_2_ samples show the particles are not only massively agglomerated, but also the particle size of samples is smaller than that of TiO_2_, which corresponds with the report of reference [54]. These results are further confirmed by TEM images (figure 9). Figure 9 shows that all samples have similar particle morphology. And the nanoparticle size of the TiO_2_ and SO_4_^2−^/TiO_2_ is around 20 nm (figure 9*a*) and 15 nm (figure 9*b–e*), respectively, which is consistent with previous report [54].

### 3.2. Catalytic activity of the catalysts for transesterification of JO

A series of solid acid catalysts including TiO_2_ and SO_4_^2−^/TiO_2_ as well as traditional liquid acid catalyst (H_2_SO_4_) for the transesterification of JO are compared, and the catalytic results of these catalysts are presented in table 2. The H_2_SO_4_ shows the highest catalytic activity and the JO conversion is 81.4%, which is attributed to a strong acidity of the homogeneous catalyst that has a full contact with the reactants and promotes the conversion of JO. However, this process shows some drawbacks of environment unfriendly, limitations of separating and difficult recycling use of catalysts. Compared with the liquid catalyst, TiO_2_ catalyst exhibits the lowest catalytic activity and the JO conversion is only 24.8%. However, the catalytic activity of the SO_4_^2−^/TiO_2_ catalysts is enhanced remarkably and the conversion of JO reaches up to above 70.9%, which is three times higher than pure TiO_2_. The reason is that the acidity and surface area of SO_4_^2−^/TiO_2_ catalysts are much higher than that of TiO_2_ (figure 5 and table 1), which can promote the transesterification of JO. Furthermore, the 1.5SO_4_^2−^/TiO_2_ catalyst reveals the highest JO conversion (73.1%) and others perform a little lower than it, because these catalysts have similar structure, surface, texture and acidic properties. Additionally, the high biodiesel selectivity (above 98%) is obtained and FAMEs composition is almost unchanged in the reaction over all catalysts. According to Scheme 1, it will see the amount and composition proportion of the various FAME products produced are as the same as the ones of JO, although the transesterification reaction is catalysed over different catalysts.

**Table 2.** Effect of different catalysts on the transesterification of JO. Reaction conditions: JO 3.0 g, methanol 1.00 g, petroleum ether 1.00 g, catalyst 0.12 g, 393 K, stirring rate 600 r.p.m., reaction time 24 h.

			FAMEs composition (%)			
catalyst	JO conversion (%)	biodiesel selectivity (%)	methyl palmitate	methyl stearate	methyl oleate	methyl linoleate
TiO_2_	24.8	98.3	14.43	7.24	42.84	29.72
0.5SO_4_^2−^/TiO_2_	72.6	99.0	14.05	7.06	44.5	31.86
1.0SO_4_^2−^/TiO_2_	71.7	98.7	14.21	6.99	44.34	31.92
1.5SO_4_^2−^/TiO_2_	73.1	98.7	14.00	6.98	44.38	32.09
2.0SO_4_^2−^/TiO_2_	70.9	98.7	14.02	6.97	44.46	31.98
H_2_SO_4_	81.4	98.2	13.79	6.85	44.56	32.39

### 3.3. Effect of various parameters over 1.5SO_4_^2−^/TiO_2_ catalyst

In order to further study systematically, the catalytic performance of the 1.5SO_4_^2−^/TiO_2_ catalyst on the transesterification of JO, the various reaction parameters (reaction time, n_CH_3___OH_ : n_JO_, catalyst content and reaction temperature) were investigated carefully. And these researches are also very useful in the production of biodiesel in industry.

#### 3.3.1. Effects of reaction time

**Figure 10.**
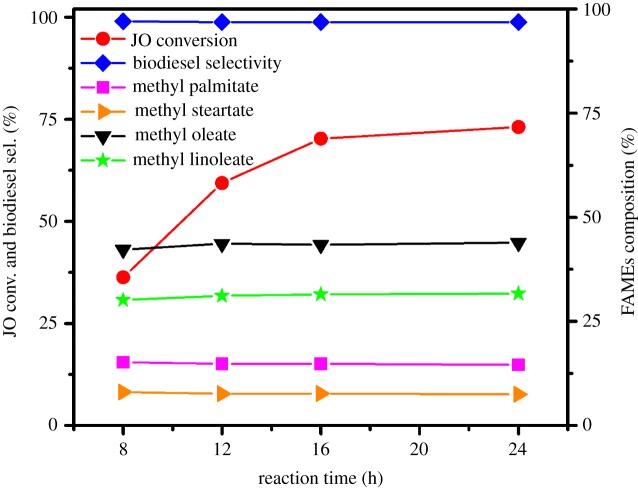
Effect of reaction time on transesterification of JO. Reaction conditions: JO 3.0 g, methanol 1.0 g, petroleum ether 1.0 g, 1.5SO_4_^2−^/TiO_2_ catalyst 0.12 g, 393 K, stirring rate 600 r.p.m.

The transesterification of JO is carried out for 8, 12, 16 and 24 h, and the results are given in figure 10. The conversion of JO increases remarkably with the increase in reaction time within 16 h. And the JO conversion increases slowly from 70.3% to 73.1% with further increasing the reaction time to 24 h. This result may be ascribed to the JO molecules being adsorbed and reacted gradually on the active sites of the solid acid catalyst along with the transesterification reaction progressed. In addition, as the reaction time increases, the selectivity to biodiesel is relatively stable and remains above 99.0%, and the composition of FAMEs is also almost unchanged (methyl palmitate = 15%, methyl stearate = 8%, methyl oleate = 44%, methyl linoleate = 31%). Thus, the time of 24 h is selected as the reaction time in the following transesterification process.

#### 3.3.2. Effects of n_CH_3___OH_ : n_JO_

**Figure 11.**
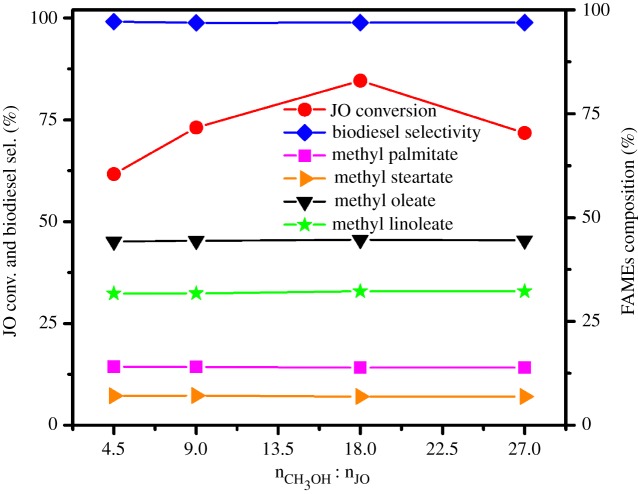
Effect of the n_CH_3___OH_ : n_JO_ on transesterification of JO. Reaction conditions: JO 3.0 g, methanol, petroleum ether 1.0 g, 1.5SO_4_^2−^/TiO_2_ catalyst 0.12 g, 393 K, 24 h, stirring rate 600 r.p.m.

The appropriate methanol content is a crucial parameter in JO transesterification that could affect the reaction rate and conversion significantly. The effect of the different molar ratio of methanol and JO from 4.5 to 27 on biodiesel production was investigated and the results are shown in figure 11. The JO conversion increases with the increase in the n_CH_3___OH_ : n_JO_ and reaches the maximum (84.6%) at the n_CH_3___OH_ : n_JO_ of 18 then begins to decrease. It is well known that the addition of methanol into the reaction mixture during the stage of transesterification could enhance the JO conversion and biodiesel selectivity [21]. However, excessive methanol results in the JO conversion decrease, which may be due to the decline of the contact probability between catalyst and reactants. This is also in good agreement with the literature reported earlier [46]. Clearly, the selectivity to biodiesel (greater than 98.8%) and FAMEs composition is almost unchanged with the rising of n_CH_3___OH_ : n_JO_.

#### 3.3.3. Effects of catalyst content

**Figure 12.**
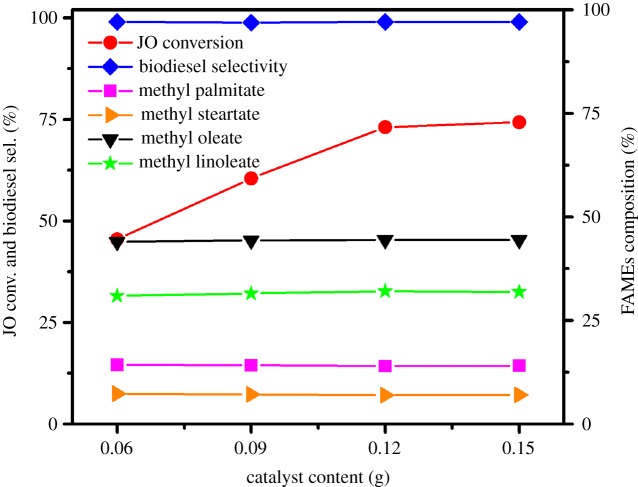
Effect of the catalyst content on transesterification of JO. Reaction conditions: JO 3.0 g, methanol 1.0 g, petroleum ether 1.0 g, 1.5SO_4_^2−^/TiO_2_ catalyst, 393 K, 24 h, stirring rate 600 r.p.m.

The results that the transesterification of JO is carried out in several different catalyst amounts (0.06, 0.09, 0.12 and 0.15 g) are shown in figure 12. As catalyst amount is increased from 0.06 to 0.12 g, the conversion of JO increases remarkably from 45.5% to 73.1%, while the X_JO_ almost remains constant even though increasing the loading amount of 1.5SO_4_^2−^/TiO_2_. This can be because the higher amounts of available catalyst allow more JO molecules to be absorbed to the catalytic active centre. Additionally, both the S_biodiesel_ and fatty acids composition have no obvious change with the increasing catalyst content. Thus, 0.12 g is the best 1.5SO_4_^2−^/TiO_2_ catalyst content for biodiesel synthesis from JO considering the low production cost.

#### 3.3.4. Effects of reaction temperature

**Figure 13.**
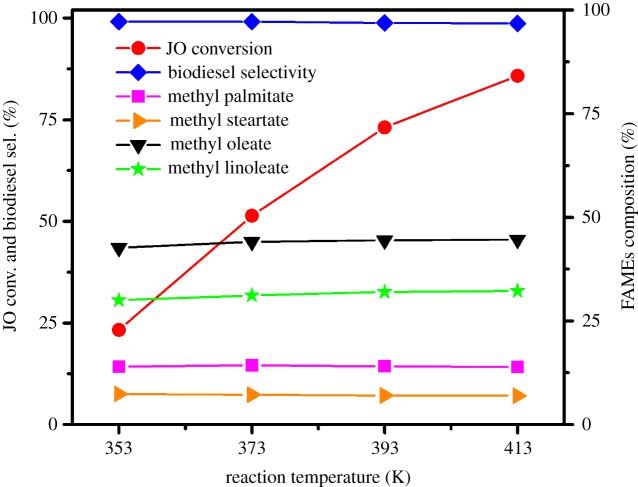
Effect of the reaction temperature on transesterification of JO. Reaction conditions: JO 3.0 g, methanol 1.0 g, petroleum ether 1.0 g, 1.5SO_4_^2−^/TiO_2_ catalyst 0.12 g, 24 h, stirring rate 600 r.p.m.

Reaction temperature is a key factor that influences biodiesel production. The effect of reaction temperature on JO conversion and selectivity to biodiesel is shown in figure 13. The conversion of JO increases remarkably from 23.3% to 85.8% with the increase in reaction temperature from 353 to 413 K. It is generally known that the catalytic activity is improved when the reaction temperature is risen, attributing to the formation of more active species in the catalyst at a higher temperature. Meanwhile, the selectivity of biodiesel remains unchanged (nearly 99.0%) as the temperature increases, and the composition and content of all fatty acids are also not found obviously different.

### 3.4. Possible transesterification reaction mechanism over SO_4_^2−^/TiO_2_ catalyst

**Figure 14.**
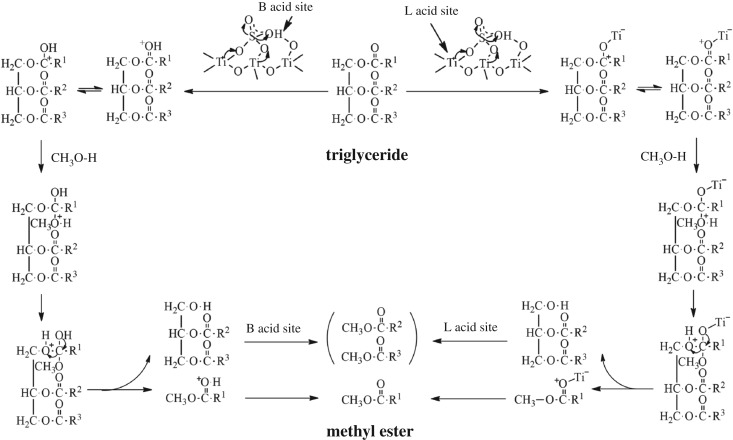
Possible mechanism of transesterification reaction over SO_4_^2−^/TiO_2_ catalyst.

The results of NH_3_-TPD and Py-IR spectra show that the SO_4_^2−^/TiO_2_ catalyst possesses many acid sites (figures 5 and 6), and it is well known that both Brønsted and Lewis acid sites are presented. Furthermore, the Brønsted acidity of the catalyst can be further enhanced by modifying its surface with sulfate groups. Furthermore, based on previous research reports on the principle of transesterification reaction catalysed by sulfated metal oxides [55,56], the possible mechanism for the transesterification of triglyceride using SO_4_^2−^/TiO_2_ catalyst is provided in figure 14.

### 3.5. Reusability behaviour of SO_4_^2−^/TiO_2_ catalyst and comparison of the solid acidic catalyst with other catalysts in the literatures

To investigate the reusability of 1.5SO_4_^2−^/TiO_2_ catalyst, a recycling experiment was conducted under the same reaction condition. After each cycle of the reaction was completed, the 1.5SO_4_^2−^/TiO_2_ particles were recovered by centrifugation and washed several times with *n*-hexane to remove any polar impurities. The washed catalyst was dried at 393 K for 8 h and re-used again in the next run. The reuse catalytic performances of 1.5SO_4_^2−^/TiO_2_ are listed in table 3. Compared with the fresh 1.5SO_4_^2−^/TiO_2_ catalyst, the selectivity of biodiesel and FAMEs composition has no significant change, although the JO conversion drops significantly from 74.8% to 27.5%, 25.7% and 25.3% in the first, second and third cycle of the catalyst, respectively.

**Table 3.** Reusability and regenerative performance of 1.5SO_4_^2−^/TiO_2_ for transesterification reaction runs. Reaction conditions: JO 3.0 g, methanol 1.00 g, petroleum ether 1.00 g, 1.5SO_4_^2−^/TiO_2_ 0.12 g, 393 K, 24 h, stirring rate 600 r.p.m.

			FAMEs composition (%)			
catalyst	JO conversion (%)	biodiesel selectivity (%)	methyl palmitate	methyl stearate	methyl oleate	methyl linoleate
fresh	73.1	98.7	14.00	6.98	44.38	32.09
first reuse	27.5	99.1	14.69	7.18	42.95	29.83
second reuse	25.7	99.1	14.75	6.95	42.51	30.14
third reuse	25.3	99.1	14.79	6.98	42.58	29.93
regenerated^a^	31.8	99.5	15.87	6.31	42.07	31.32

^a^The regenerated 1.5SO_4_^2−^/TiO_2_ is obtained from used 1.5SO_4_^2−^/TiO_2_ by calcining at 823 K for 3 h.

**Figure 15.**
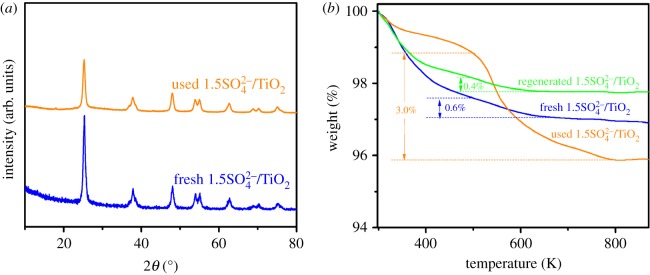
XRD diffraction patterns (*a*) and TG curves (*b*) of the fresh and used 1.5SO_4_^2−^/TiO_2_ catalysts.

In order to further explore the causes of the descending activity of the used catalyst in JO transesterification reaction. The fresh and used 1.5SO_4_^2−^/TiO_2_ catalysts are characterized by the methods of N_2_-adsorption, NH_3_-TPD, XRD and TG. The XRD diffraction patterns have confirmed that the structure destruction of used 1.5SO_4_^2−^/TiO_2_ did not occur (figure 15*a*). However, as shown in table 1, compared with the fresh catalyst, the S_BET_ and *V*_Mesopore_ of used catalyst decrease obviously from 76.4 m^2^ g^−1^ and 0.26 cm^3^ g^−1^ to 62.1 m^2^ g^−1^ and 0.18 cm^3^ g^−1^, respectively. And the TG analyses also show that the weight loss of the used catalyst (3.0%) is much greater than that of the fresh catalyst (0.6%), indicating that some carbonaceous residues (cokes) are formed on the used catalyst (figure 15*b*). It is well known that deposits of cokes on the catalyst surface can cover the active centre and block the mesopore channels [57,58]. So the catalytic activity of the used catalyst decreases significantly. Additionally, the regenerated 1.5SO_4_^2−^/TiO_2_ is obtained by calcining at high temperature, which successfully removes these cokes on the surface of the used 1.5SO_4_^2−^/TiO_2_, being verified sufficiently by the results of TG analysis. However, compared with the used catalyst (27.5%), the JO conversion merely rises slightly over a regenerated catalyst (31.8%). In other words, the deposition of coke is unlikely to be a major reason for the deactivation of SO_4_^2−^/TiO_2_ catalyst in this reaction. On the other hand, the result of NH_3_-TPD analysis (table 1) shows that the total NH_3_ desorbed of the used 1.5SO_4_^2−^/TiO_2_ (0.24 mmol g^−1^) is much less than that of the fresh 1.5SO_4_^2−^/TiO_2_ (0.39 mmol g^−1^), which means the acidity and activity of the catalyst reduces significantly after being used in transesterification reaction. The reason is that conventional preparation of SO_4_^2−^/TiO_2_ is *via* a post-synthesis grafting method, which leads to relatively weak interactions between SO_4_^2−^ and TiO_2_ framework and thereby poor catalytic stability, and the leaching of sulfate group also easily happens from SO_4_^2−^/TiO_2_ catalyst in a solvent system for a long-running process [4,59,60]. Consequently, the 1.5SO_4_^2−^/TiO_2_ catalyst shows poor catalytic stability and deactivates easily in the transesterification of JO, attributing to leaching of SO_4_^2−^ and blocking of the active sites by carbonaceous residues deposition. Therefore, the approaches of improving the reusability of sulfated TiO_2_ need to be developed by the researchers in the future.

Table 4 compares the catalytic performance of 1.5SO_4_^2−^/TiO_2_ with the solid acidic catalysts reported by other researchers for transesterification of JO. The results show that the high biodiesel yield of 85.3% obtained in this study was obtained under much milder reaction condition and less CH_3_OH dosage and catalyst loading. Although slightly higher biodiesel yields (greater than 92%) were reported by other literature, these transesterification processes generally require much higher reaction temperature and pressure. Therefore, the process puts forward a higher requirement for reaction equipment and has an unsatisfactory economy. In addition, Raia *et al*. [19] reported that the production of biodiesel from JO can be realized at the similar reaction temperature and atmospheric pressure, but the low yield of biodiesel (59.4%) was obtained even though the amount of the catalyst reached 10 wt%. This result therefore shows the superiority of the findings in this work in producing biodiesel with high quality from JO.

**Table 4.** Comparison of solid acidic catalysts for biodiesel production from JO.

	this work	Suzuta *et al*. [61]	Raia *et al*. [19]	Pan *et al*. [62]	Meloni *et al*. [22]
feedstock	JO	JO	JO	JO	JO
catalyst	1.5SO_4_^2−^/TiO_2_	FeO*_x_*/SiO_2_	SO_4_^2−^/ZrO_2_	MPD-SO_3_H-IL	Al-SBA-15 (22)
CH_3_OH:JO (mol)	9	218	10	50	12
catalyst content (wt%)	4	15	10	6	8
reaction pressure (MPa)	0.2	1	AP^a^	1.4	4
reaction temperature (°C)	140	220	150	160	180
reaction time (h)	24	3	8	8	24
biodiesel yield (%)	85.3	99.0	59.4	94	92

^a^AP represents atmospheric pressure.

## 4. Conclusion

An efficacious and heterogeneous approach to produce the biodiesel from JO catalysed by SO_4_^2−^/TiO_2_ catalysts has been developed. The TiO_2_ and SO_4_^2−^/TiO_2_ catalysts were successfully synthesized and characterized and used in the transesterification of JO. The results demonstrate that the new Brønsted acid sites are formed and the crystalline structure is not destroyed after treating with sulfuric acid of TiO_2_ catalyst. And the acid intensity, BET surface area and mesoporous volume of SO_4_^2−^/TiO_2_ catalysts are stronger than that of TiO_2_ catalyst. Furthermore, the catalytic activity tests show that the SO_4_^2−^/TiO_2_ catalysts exhibit higher catalytic activity than TiO_2_ catalyst, attributed to the larger surface area, smaller particle size and stronger acidity. Then the effects of operation conditions were obtained by testing of catalytic activity under the different reaction conditions (time, CH_3_OH : JO, catalyst content and temperature). In addition, the reusability and deactivation behaviour of 1.5SO_4_^2−^/TiO_2_ was further studied. Combined with the experiment and characterization results of the fresh and used catalysts, the cause for a decrease in the catalyst activity is due to a combination of SO_4_^2−^ leaching and the blocking of the active sites by carbonaceous residues deposition. These researches are very useful in the scaling-up of this sustainability transesterification process for the production of biodiesel from low-cost feedstocks.

## Data accessibility

We include all the experimental data in the electronic supplementary material, which are available at https://dx.doi.org/10.5061/dryad.ds6p94f [63].
